# Benign Spindle Cell Lesion of the Nasal Cavity: A Rare Presentation of Solitary Fibrous Tumor

**DOI:** 10.7759/cureus.60220

**Published:** 2024-05-13

**Authors:** S Vijayasundaram, Venkataramani Agathiyanathan, Padmanabhan Karthikeyan, Kirubhagaran Ravichandran

**Affiliations:** 1 Otolaryngology - Head and Neck Surgery, Mahatma Gandhi Medical College and Research Institute, Pondicherry, IND

**Keywords:** diagnostic nasal endoscopy, excision, carcinoma, lesion, nasal cavity, spindle cell

## Abstract

Solitary fibrous tumors (SFTs) are rare neoplasms primarily found in the pleural region but have been documented in diverse extrapleural sites, including the nasal cavity and paranasal sinuses, albeit infrequently. Here, we present a case of a 48-year-old female who presented with a right-sided nasal mass and associated ophthalmologic symptoms, ultimately diagnosed with a benign spindle cell lesion localized to the nasal cavity. The patient underwent a comprehensive evaluation involving clinical examination, radiological imaging, and histopathological analysis, leading to the identification of a benign solitary fibrous tumor. Notably, diagnosing SFTs in the nasal cavity presents challenges due to their nonspecific clinical and imaging features, necessitating a multidisciplinary approach for accurate diagnosis and optimal management. Surgical excision, preferably via endoscopic techniques, remains the cornerstone of treatment based on tumor characteristics and extent. This case underscores the importance of recognizing uncommon presentations of sinonasal lesions, navigating diagnostic complexities, and emphasizing the critical role of multidisciplinary collaboration in achieving favorable treatment outcomes for patients with such nasal cavity tumors.

## Introduction

Solitary fibrous tumors (SFTs) are rare neoplasms that usually arise in the pleural region. Initially, these tumors were identified as pleural neoplasms originating from spindle cells. They are also categorized as a benign form of mesothelial tumor. Previously, there have been several reports of SFTs occurring in various sites beyond the pleura, including the liver, parapharyngeal space, sublingual glands, tongue, parotid gland, thyroid, periorbital region, but rarely in the nose and paranasal sinus area [1].

SFTs that affect the nasal cavity are exceptionally uncommon. To the best of our knowledge, less than 40 cases have been documented in the existing literature [1, 2]. The clinical and imaging characteristics of SFTs are nonspecific, and they have a wide range of differential diagnoses. This report describes a rare case of benign spindle cell lesion of the nasal cavity and paranasal sinuses.

## Case presentation

A patient in her late 40s presented to the OPD with complaints of right-sided nasal obstruction for three months and right eye blurring of vision for one month. She also complained of excessive tearing in the right eye, along with difficulty in fully closing the eye and weakness in the right eyelid. There were no complaints of nasal discharge, nasal bleed facial pain, facial puffiness, cheek swelling, post nasal drip, heaviness of the head ear pain, ear discharge, hard of hearing fever, or throat pain. The patient reportedly underwent surgery to remove a mass in the left breast six years ago, although detailed reports of the procedure were not available.

During the examination of the nose, anterior rhinoscopy revealed a grossly deviated nasal septum to the right, which obscured visualization of the nasal mass. However, the left nasal cavity appeared clear upon inspection. Additionally, the cold spatula test indicated decreased fogging on both sides, with the right side demonstrating more pronounced impairment compared to the left.

**Figure 1:**
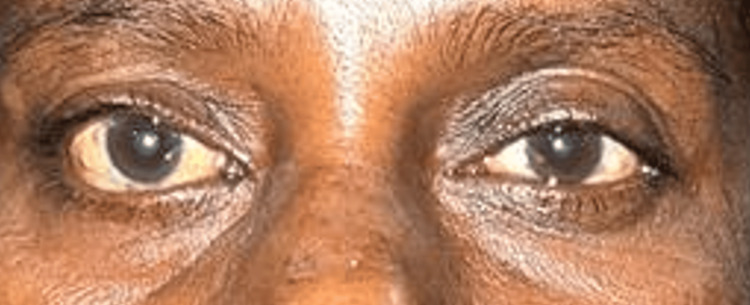
Right eye proptosis - forwards and outwards

In terms of eye findings, the patient exhibited proptosis of the right eye, which was noted to protrude forward and outwards (Figure 1). Both eyes demonstrated a full range of movements during the examination. Visual field testing revealed the presence of multiple defects primarily located in the inferior quadrant of the right eye.

Investigations

**Figure 2:**
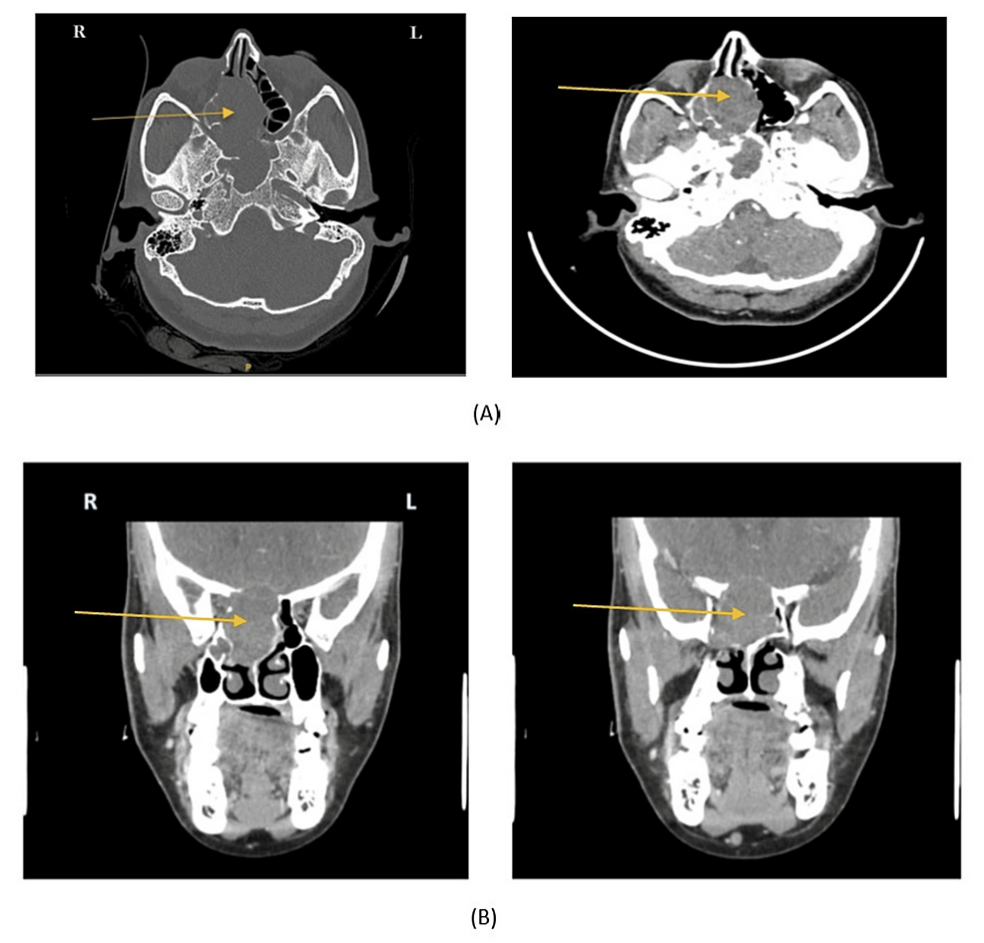
CT scan of the paranasal sinus (A) CT of the paranasal sinuses-axial section showing a soft tissue density that is hypodense with that of the brain and a contrast CT of the same section showing mild fairly uniform contrast enhancement. (B) Contrast CT of Paranasal sinuses-coronal view showing a soft tissue density occupying the Right ethmoid sinus and extending into the right sphenoid sinus, the nasal septum, and possibly intracranially.

A 0-degree diagnostic nasal rigid scope examination revealed a grossly deviated nasal septum towards the right and a bulge was appreciated at the posterior end of the left side of the nasal septum. A CT scan of the paranasal sinus showed a heterogenous and hypodense lesion, isodense with that of the brain. The lesion arose from the right ethmoidal sinus and extended into the sphenoid sinus causing compression on right optic canal structures extending towards the nasal septum and intracranially (Figure 2).

**Figure 3:**
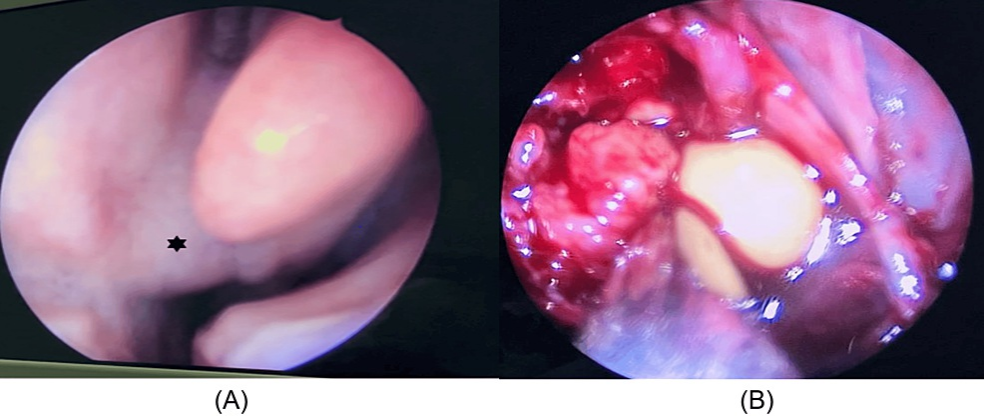
Intra-operative findings A) A posterior septal bulge caused by the mass seen in the left nasal cavity (B) Firm friable mass arising from the right posterior ethmoid and right sphenoid sinus pushing the left nasal cavity and eroding the posterior end of the nasal septum. Yellowish purulent material was noted arising from the right sphenoid sinus.

Considering the expansion extent and erosion, we came up with a diagnosis of ethmoido-sphenoidal mucocele. Functional endoscopic sinus surgery was performed, multiple nasal-mass biopsies were taken and near-complete disease clearance was given (Figure 3).

The specimens were taken for biopsy as multiple greyish-white fragments and sent for histopathological examination which showed features suggestive of benign spindle cell soft tissue tumor, due to CD34 positivity, with a possibility of a benign solitary fibrous tumor to be considered.

Microscopy

**Figure 4:**
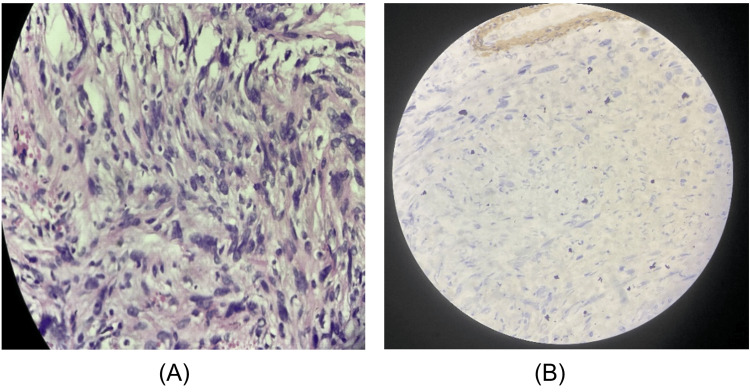
Histopathological findings (A) The section studied showed benign tumor cells composed of ovoid to spindle-shaped cells arranged in interlacing sweeping fascicles and focal vague storiform patterns. (B) Immunohistochemistry for CD34 was positive (a tumor marker for solitary fibrous tumor).

The section studied showed benign tumor cells arranged in interlacing sweeping fascicles and focal vague storiform patterns. The specimen was then subjected to immunohistochemistry and found to have a CD34 positivity further solidifying the diagnosis of a solitary fibrous tumor (Figure 4).

**Figure 5:**
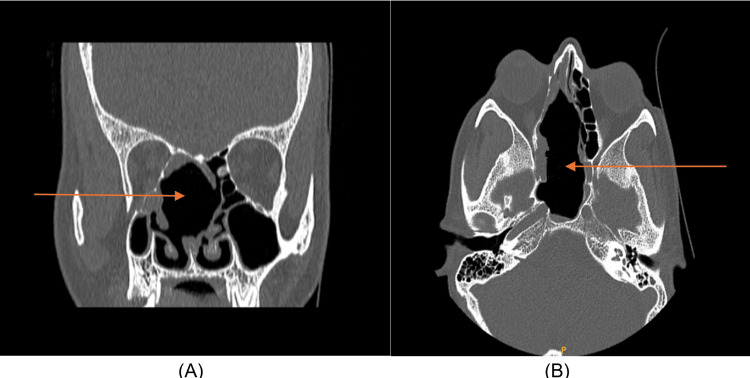
Postoperative CT of the Paranasal sinuses after 10 days (A) Coronal section reveals the absence of mass in the previously occupied right posterior ethmoid and right sphenoid sinuses. (B) Axial view showing a mid-nasal septal defect post septoplasty, sphenoidotomy, and nasal mass excision.

On follow-up examination, the B/L nasal cavities were clear and patent and a postoperative CT of the paranasal sinuses was done (Figure 5).

The patient had an improvement in her right eye field of vision with visual field charting showing a reduction in visual field defects. There was a clinically apparent progressive regression in her right eye proptosis. The patient has regular follow-up and is doing well so far.

## Discussion

Solitary fibrous tumors (SFTs) are uncommon tumors that arise from mesothelial cells and typically manifest in adults in their third to fourth decade of life. However, the age of onset has been reported to range from nine to 86 years old [3]. Traditionally, SFTs were thought to originate from spindle cells in the pleura. However, they have also been documented in several extra-pleural locations, including the liver, parapharyngeal space, sublingual gland, tongue, orbit, eyelids, nose, paranasal sinuses, parotid gland, thyroid, and laryngopharynx [4-6].

SFTs located in the extrathoracic region of the head and neck region also tend to exhibit a benign behavior that is similar to their intrathoracic counterparts [7]. SFTs when located in the nasal cavity and extra-pleural sites are typically benign, in contrast to the more aggressive behavior observed in 23% of pleural SFTs. Nevertheless, malignant transformation in SFTs is rare, and there have been no reports of such transformation occurring in the sinonasal region until now [8].

Sinonasal SFTs as in our case typically manifest as a painless mass that grows slowly. When symptomatic, the most common presentation of SFT is nasal obstruction, rhinorrhea, intermittent epistaxis, and exophthalmos. Based on the available literature, the size of the tumor typically ranges from 2.8 to 8 cm in its major axis [9, 10]. Typically, SFTs are encapsulated, reddish, and fibrous in appearance. In this case, a soft to firm, well-encapsulated mass was observed. Non-contrast CT imaging revealed homogeneous isoattenuation compared to gray matter, and these tumors typically exhibit significant enhancement after the administration of contrast material [10]. Typically, on T2-weighted MRI, the soft-tissue component of SFTs appears iso- to hypo-intense [11].

SFTs are generally highly vascular, which results in marked enhancement after the administration of contrast material. Depending on the size of the tumor, it may cause deviation of the nasal septum, remodeling of bone structures, local absorption, and even reactive sclerosis [10]. In rare cases, SFTs may extend into the orbit and cranial cavity, potentially passing through the cribriform plate and ethmoid roof [1].

On MRI, SFTs typically appear homogeneously isointense to gray matter on T1-weighted images, while on T2-weighted images they generally exhibit a heterogeneous isointense or hypointense appearance. The predominant low signal on T2-weighted images is a characteristic feature of these tumors, but it is not specific to them [10]. Although the low signal on T2-weighted images is not specific to SFTs, the combination of this feature with significant enhancement after gadolinium injection is highly predictive of SFTs [12, 13].

Due to the variety of clinical presentations and the lack of specific imaging features, the clinical differential diagnosis of nasal cavity SFTs should primarily consider fibrosarcoma, hemangiopericytoma, and nasopharyngeal carcinoma [14]. SFTs have specific macroscopic, histologic, and immunophenotypic characteristics that are helpful for the pathologist to make a correct diagnosis. These features are considered to be pathognomonic for SFTs [15]. Macroscopically, SFTs are usually pedunculated or sessile and encapsulated masses. Histologically, SFTs are composed of spindle cells arranged in a nonspecific pattern with varying vascularity as was observed in this case. SFT is typically characterized by the presence of regions of hyalinization in close proximity to deposits of collagen [7].

Typically, immunohistochemical analysis of SFT reveals positivity for CD34 and vimentin (Figure 3), which is a common finding. Additionally, more than 50% of SFT tumors also exhibit positivity for CD99 [7]. Studies using electron microscopy and immunohistochemistry have indicated that mesenchymal cells with a fibroblast-like appearance are the main source of SFTs [16]. The diagnosis of SFT is based on identifying the unique features of the tissue under a microscope and specific immunohistochemical markers [10].

When it comes to SFTs located in the nasal cavity, endoscopic excision is the most preferred surgical method. However, other approaches such as lateral rhinotomy, medial maxillectomy, external ethmoidectomy, and transfacial endoscopic approaches have also been reported [17, 18].

## Conclusions

In conclusion, SFTs are rare neoplasms that can occur in extra-pleural sites such as the nasal cavity and paranasal sinuses. This case emphasizes the diagnostic challenges posed by SFTs due to their nonspecific clinical and imaging characteristics. While typically benign, SFTs in the nasal cavity require prompt surgical intervention due to their potential for local invasion.

Radiological imaging aids in diagnosis, but histopathological examination, with characteristic immunohistochemical markers like CD34 and vimentin, is crucial for definitive diagnosis. Treatment primarily involves surgical excision, often through endoscopic methods. This underscores the importance of a multidisciplinary approach for accurate diagnosis and management of nasal cavity SFTs.
